# Modeling combination chemo‐immunotherapy for heterogeneous tumors

**DOI:** 10.1002/qub2.98

**Published:** 2025-03-14

**Authors:** Shaoqing Chen, Zheng Hu, Da Zhou

**Affiliations:** ^1^ School of Mathematical Sciences Xiamen University Xiamen China; ^2^ National Institute for Data Science in Health and Medicine Xiamen University Xiamen China; ^3^ Key Laboratory of Quantitative Synthetic Biology Shenzhen Institute of Synthetic Biology Shenzhen Institute of Advanced Technology Chinese Academy of Sciences Shenzhen China

**Keywords:** chemo‐immunotherapy, immune checkpoint blockade, mathematical modeling, neoantigen heterogeneity, treatment strategy

## Abstract

Hypermutable cancers create opportunities for the development of various immunotherapies, such as immune checkpoint blockade (ICB) therapy. However, emergent studies have revealed that many hypermutated tumors have poor prognosis due to heterogeneous tumor antigen landscapes, yet the underlying mechanisms remain poorly understood. To understand the mechanisms that govern the responses to therapies, we develop mathematical models to explore the impact of combining chemotherapy and ICB therapy on heterogeneous tumors. Our results uncover how chemotherapy reduces antigenic heterogeneity, creating improved immunological conditions within tumors, which, in turn, enhances the therapeutic effect when combined with ICB. Furthermore, our results show that the recovery of the immune system after chemotherapy is crucial for enhancing the response to chemo‐ICB combination therapy.

## 1 INTRODUCTION

Immunogenic tumors can elicit immune responses that suppress tumor growth [1]. Hypermutated cancers produce large amounts of neoantigens and are often considered highly immunogenic and responsive to immunotherapies [2, 3]. Immune checkpoint blockade (ICB) therapy has shown promising results in improving the survival of patients with various types of malignant tumors [4, 5]. Recent evidence suggests that intra‐tumoral heterogeneity has a significant impact on immunotherapy prognosis [6, 7, 8]. Immunogenic tumor antigens do not lead to tumor rejection when the fraction of cells bearing them is low [9, 10]. Cancers, characterized by diverse neoantigens, can be unresponsive to ICB.

Immune escape has been confirmed as one of the emerging hallmarks of cancer [11]. Immune escape in hypermutable tumors is assisted by checkpoint ligands overexpression to maintain the imbalance between immune surveillance and cancer growth [12]. Checkpoint antibody inhibitors, such as anti‐PD‐1/PD‐L1, are a novel class of inhibitors that can lead to tumor suppression via modulation of tumor‐immune interactions [13]. The effect of ICB treatment is dependent on the auto‐immunity to eliminate cancers. With a heterogeneous neoantigen landscape, the negative selection imposed on tumor antigens can be dependent on their frequencies [9, 10]. Therefore, intra‐tumoral antigen heterogeneity can lead to compromised immune predation and ICB therapy response [14, 15]. Meanwhile, chemotherapy faces a similar treatment challenge. The presence of intra‐tumoral heterogeneity and the resulting variability in patients can lead to resistance against chemotherapy [16, 17].

To tackle the challenge posed by intra‐tumoral heterogeneity, the development of combination protocols involving chemotherapy and immunotherapy holds promise as a strategy for effective cancer treatment [18, 19, 20]. Preliminary clinical studies have indicated enhanced effectiveness when immunotherapy is administered alongside chemotherapy [21]. Although chemotherapy is traditionally viewed as immunosuppressive, emerging data suggest that it may also harbor immunostimulatory properties [22, 23]. This dual nature presents the potential to create a favorable immunogenic condition within the tumor, a feat challenging to achieve solely through immunotherapy.

Tumor heterogeneity has also fueled investigations into optimal and adaptive therapy through mathematical modeling [16, 24, 25, 26, 27]. Nevertheless, further exploration is essential not only to identify the most suitable partners for ICB but also to determine the optimal regimen for combination chemo‐immunotherapy [18, 19, 28]. Understanding the governing mechanisms of response to combination therapy is crucial for optimizing treatment strategies, which remains incompletely understood. Theoretical models specifically considering neoantigen heterogeneity in chemo‐ICB combination therapy are currently very lacking.

In this study, we aim to characterize the evolving dynamics of antigenically heterogeneous tumors under intrinsic immune selection and various therapies. Through mathematical models, we seek to unveil the unique evolutionary patterns of tumors responding to different therapeutic interventions, providing crucial insights for the design of future combination therapies. Existing literature suggests that tumor cell populations suitable for ICB treatment may exhibit resistance to chemotherapy [29, 30]. Immune cold tumors typically show limited response to ICB monotherapy, but these tumors often display heightened expression of receptors promoting vasculogenesis and angiogenesis, such as vascular endothelial growth factor [31, 32]. To transform these immune cold tumors into inflamed ones, combination therapy involving diverse treatment modalities, including chemotherapy, emerges as a promising therapeutic strategy. These approaches are actively under investigation [18, 32]. Our focus is on uncovering the distinct mechanisms underlying these dynamics and the intra‐tumoral antigenic profile of tumors. We develop an immunogenic heterogeneity model (IHeM) to explore the impact of combination therapy on heterogeneous tumors. For comparison, we also establish a typical immunogenic homogeneity model (IHoM) for antigenically homogeneous tumors. We observe that heterogeneous tumors demonstrate resistance to ICB therapy but exhibit early remission when treated with chemotherapy alone. It is worth noting that the combination of chemotherapy and ICB demonstrates a curative effect on heterogeneous cancers modeled by the IHeM. Our results also reveal that chemotherapy effectively mitigates antigen heterogeneity, enhancing the immunological conditions within cancers and thereby improving the efficacy of combination therapy. Furthermore, our findings highlight that the recovery of the immune system significantly enhances the response to combination therapy.

## 2 RESULTS

### 2.1 Modeling cancer evolution under immune system selection

We consider two distinct models: the IHoM and the IHeM. These models are designed to capture the evolutionary dynamics of antigenically homogeneous and heterogeneous tumors, respectively. In order to represent the interactions between cancer cells and the immune system, we employ the predator–prey framework, as described in Ref. [33]. In this framework, cancer cells are akin to prey, susceptible to being targeted or attacked by immune cells, which act as predators in the biological system.

**FIGURE 1:**
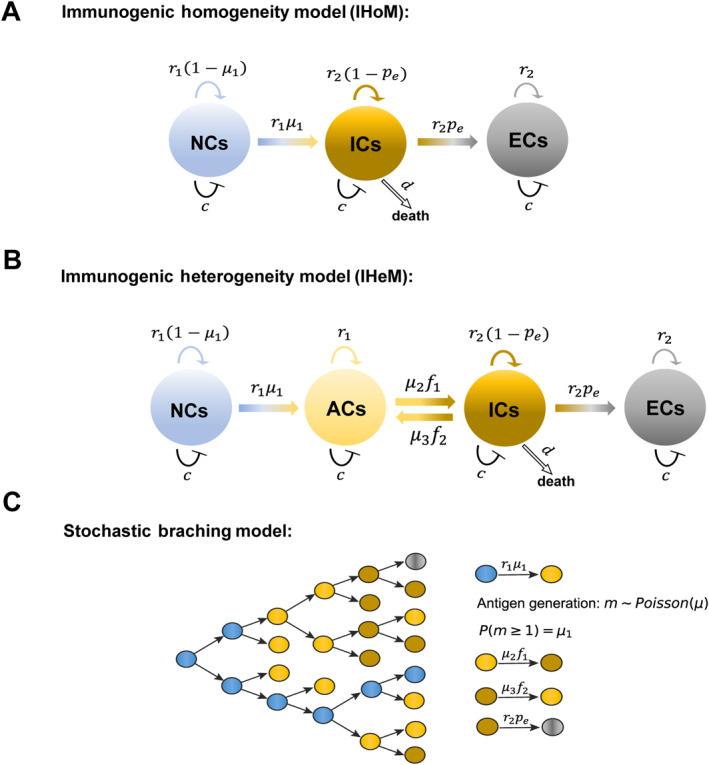
Representation of our models. (A) Schematic representation of the IHoM. Different compartments represent different cell immunogenic status. From left to right, it represents NCs, ICs and immune ECs. For each NC, it can divide at rate $r_{1}$, and then either give birth to two identical offsprings with birth rate $1\;–\;\mu_{1}$ or transform into one IC at rate $\mu_{1}$. For each IC, it can divide at rate $r_{2}$, and then give birth to two identical offsprings at rate $1\;–\;p_{e}$ or transform into an EC at rate $p_{e}$. ICs die at rate d. (B) Schematic representation of the IHeM. From left to right, it represents NCs, ACs, ICs and immune ECs. An AC transforms into an IC at rate $\mu_{2}f_{1}$. ICs transform into ACs at rate $\mu_{3}f_{2}$. Other cellular processes are similar to the ones of the IHoM represented in A. (C) Schematic presentation of the stochastic branching model. The blue filled circles represent NCs, yellow filled circles represent ACs, blown filled circles represent ICs and gray filled circles represent ECs. ACs, antigenic cells; ECs, escaped cells; ICs, immunogenic cells; IHeM, immunogenic heterogeneity model; IHoM, immunogenic homogeneity model; NCs, neutral cells.

In the IHoM, it is assumed that immunogenic cells (ICs) originate from antigenically neutral cells (NC) through the accumulation of antigens. Our model involves a cell population organized into three compartments, each representing a distinct cell type. As illustrated in Figure 1A, compartment NCs represents antigenically NCs, compartment ICs represents ICs, and compartment ECs represents immune‐escaped cells. For NCs, we assume a division rate of $r_{1}$. Upon division, a cell can either undergo transformation into one IC through the accumulation of antigenic mutations at a rate of $\mu_{1}$ or produce two identical offspring at a rate of $1\;–\;\mu_{1}$. ICs divide at a rate of $r_{2}$ and face a death rate of d due to immune system activity. Through the overexpression of immune checkpoint ligands, cancer cells can evade immune elimination of ICs. Additionally, to account for ICB therapy, we introduce immune escape [9, 34], wherein ICs can transform into immune‐escaped cells (ECs) at a rate of $p_{e}$ (Figure 1A). The IHoM delineates the fundamental mechanisms governing tumor evolution under immune system selection. The cellular processes in the IHoM are summarized as follows:

(a) NC $\overset{r_{1}\left(1-\mu_{1}\right)}{\to }$ NC + NC,
(b) NC $\overset{r_{1}\mu_{1}}{\to }$ IC,
(c) IC $\overset{r_{2}\left(1-p_{e}\right)}{\to }$ IC + IC,
(d) IC $\overset{d}{\to }\emptyset$,
(e) IC $\overset{r_{2}p_{e}}{\to }$ EC,
(f) EC $\overset{r_{2}}{\to }$ EC + EC.

In the IHeM, we integrate negative frequency‐dependent selection into the IHoM [10, 35], as depicted in Figure 1B. Cells in compartment NCs can initially transform into antigenic cells (ACs) through antigen accumulation. ACs can proliferate at a rate of $r_{1}$ and remain inconspicuous to the immune system when present in low frequencies [9, 10]. A transition from ACs to ICs occurs with a rate of $\mu_{2}f_{1}$, where $f_{1}$ denotes the frequency of ACs, that is, the ratio of the number of ACs to the sum of NCs and ACs. To model the impact of tumor antigen heterogeneity on immune recognition ineffectiveness, we assume that ICs can revert to ACs at a rate of $\mu_{3}f_{2}$, where $f_{2}$ represents the ratio of the number of ICs to the sum of ICs, NCs, and ACs. Neutral and ICs in the IHeM proliferate and undergo cell death similar to the IHoM. The cellular processes in the IHeM are summarized as follows:

(a) NC $\overset{r_{1}\left(1-\mu_{1}\right)}{\to }$ NC + NC,
(b) NC $\overset{r_{1}\mu_{1}}{\to }$ AC,
(c) AC $\overset{r_{1}}{\to }$ AC + AC,
(d) AC $\overset{\mu_{2}f_{1}}{\to }$ IC,
(e) IC $\overset{r_{2}\left(1-p_{e}\right)}{\to }$ IC + IC,
(f) IC $\overset{\mu_{3}f_{2}}{\to }$ AC,
(g) IC $\overset{d}{\to }\emptyset$,
(h) IC $\overset{r_{2}p_{e}}{\to }$ EC,
(i) EC $\overset{r_{2}}{\to }$ EC + EC.

Moreover, competitive interactions between different cells are also considered in our model [36, 37]. We assume that cell competition causes one of the two competing cells to die at equal chance. For example, NC + NC $\overset{c}{\to }$ NC describes the competition between two NCs, and NC + AC $\overset{c}{\to }$ NC describes the competition between NC and AC. Here c is the competition death rate that characterizes the competition strength. Processes NC $\overset{r_{1}\mu_{1}}{\to }$ IC and IC $\overset{r_{2}p_{e}}{\to }$ EC are used to describe accumulation of antigenic mutations and immune escape. Considering the processes of asymmetric cell division (e.g., NC → NC + IC) and symmetric cell division (e.g., NC → IC + IC) both characterize the transition of one cell type to another, we use NC $\overset{r_{1}\mu_{1}}{\to }$ IC to keep the model as minimum as possible (see METHODS).

Based on the above assumptions, we generate a stochastic master Equation (10) (see METHODS) and derive model equations for IHoM, as shown below:

(1) $$\left\{\;\begin{array}{l} \frac{dN}{dt}=r_{1}\left(1-\mu_{1}\right)N-r_{1}\mu_{1}N-c\left(N^{2}+IN+EN\right),\\ \frac{dI}{dt}=\left(r_{2}-r_{2}p_{e}\right)I+r_{1}\mu_{1}N-r_{2}p_{e}I-dI-c\left(I^{2}+NI+EI\right),\\ \frac{dE}{dt}=r_{2}E+r_{2}p_{e}I-c\left(E^{2}+NE+IE\right).\; \end{array}\right.$$

Here, N, I, and E denote the quantities of NCs, ICs, and ECs, respectively. Neoantigen generation is linked to cell division and is expressed as $r_{1}\mu_{1}N$. The processes of cell death and immune escape in ICs are represented by dI and $r_{2}p_{e}I$, respectively. Cell competition processes are delineated by terms such as $c(N^{2}+IN+EN\;)$ at the end of each equation.

For the IHeM, the stochastic dynamics of IHeM are captured by the master Equation (11) (see METHODS). Similarly, we derive model Equation (2) for IHeM according to Equation (11) as follows:

(2) $$\left\{\begin{array}{l} \frac{dN}{dt}=r_{1}\left(1-\mu_{1}\right)N-r_{1}\mu_{1}N-c\left(N^{2}+AN+IN+EN\right),\\ \frac{dA}{dt}=r_{1}A+r_{1}\mu_{1}N-\mu_{2}f_{1}A+\mu_{3}f_{2}I-c\left(A^{2}+NA+IA+EA\right),\\ \frac{dI}{dt}=\left(r_{2}-d\right)I-\mu_{3}f_{2}I+\mu_{2}f_{1}A-{2r}_{2}p_{e}I-c\left(I^{2}+NI+AI+EI\right),\\ \frac{dE}{dt}=r_{2}E+r_{2}p_{e}I-c\left(E^{2}+NE+AE+IE\right).\; \end{array}\right.$$

Here, N, A, I, and E denote the quantities of NCs, ACs, ICs, and ECs, respectively. Neoantigen generation is linked to cell division and is expressed as $r_{1}\mu_{1}N$. The transitions between ACs and ICs, under negative frequency‐dependent selection, are characterized by $\mu_{2}f_{1}A$ and $\mu_{3}f_{2}I$ in the second and third equations, respectively. The processes of cell death and immune escape in ICs are represented by dI and $r_{2}p_{e}I$, respectively. Cell competition processes are delineated by terms such as $c\left(N^{2}+AN+IN+EN\right)$ at the end of each equation.

### 2.2 Modeling ICB therapy and chemotherapy

**FIGURE 2:**
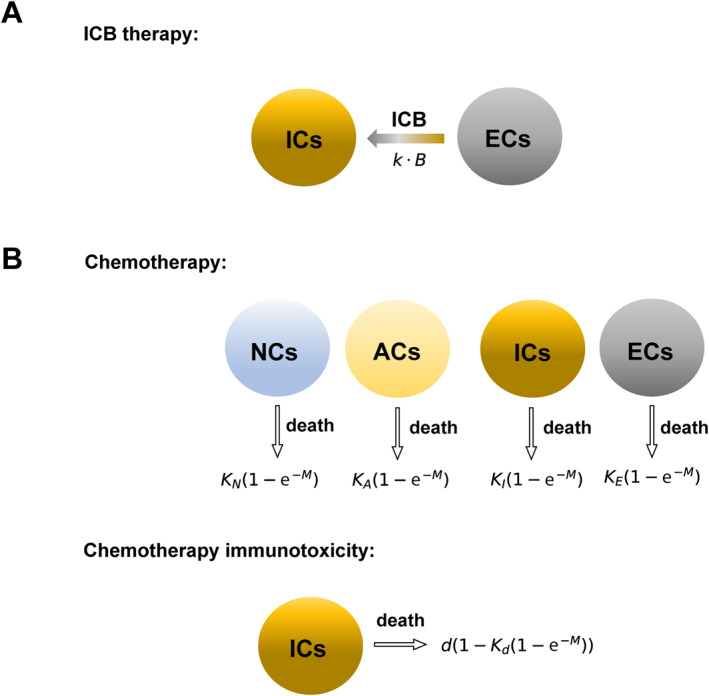
Representation of ICB and chemotherapy models. (A) Schematic representation of ICB therapy. Here B represents the blood concentration of ICB drugs. ICs represent ICs and ECs represent immune escaped cells. (B) Schematic representation of chemotherapy. Here M represents the blood concentration of chemotherapy drugs. NCs represent NC, ICs represent ICs, and ECs represent immune escaped cells. The parameters $K_{N}$, $K_{A}$, $K_{I}$, and $K_{E}$ denote the sensitivity of each cell type to chemotherapy. For example, the death rate of neutral cancer cells under chemotherapy is expressed as $K_{N}\;\left(1\;–\;e^{-M}\right)$. The parameter $K_{d}$ represents chemotherapy‐induced damage to the immune system (chemotherapy immunotoxicity), modeled by adjusting the death rate as $d\;\to \;d\;\cdot \;\left(1\;–\;K_{d}\left(1\;–\;e^{-M}\right)\right)$. ICB, immune checkpoint blockade; ICs, Immunogenic cells; NCs, Neutral Cells.

We model the impact of therapies and the underlying mechanisms governing therapy response based on our IHoM and IHeM. In various cancer types, ICB therapy has shown enhanced survival for a subset of patients [4, 5]. The effect of ICB is represented in our model by reverting immune‐escaped cells back to ICs (Figure 2A). To model therapies, we employ impulsive differential equations [33, 38] as follows:

(3) $$\left\{\begin{array}{c} \frac{dM}{dt}=-\gamma M+v_{m}\left(t\right),\\ \frac{dB}{dt}=-\gamma B+v_{b}\left(t\right),\\ v_{m}\left(t\right)=v_{m}\cdot sgn{\left(\sin\left(f\cdot t\right)\right)}^{+},\\ v_{b}\left(t\right)=v_{b}\cdot sgn{\left(\sin\left(f\cdot t\right)\right)}^{+}, \end{array}\right.$$

where M and B represent the blood concentration of chemotherapy and ICB therapy drugs, respectively. γ is the drug decay rate, $v_{m}$ and $v_{b}$ are the dose of every pulse of drug delivery. In addition,

(4) $$sgn{\left(x\right)}^{+}=\left\{\begin{array}{c} 1,\;x>0\\ 0,\;x\leq 0 \end{array}\right.\;.$$

The parameter f governs the therapy cycle, and we define T as T=2π/f, representing the duration of each therapy cycle. During each ICB therapy pulse, a portion (k·B) of ECs transforms into ICs based on the drug concentration B. Consequently, the ODEs describing ICB therapy for the IHoM are given as follows:

(5) $$\left\{\;\begin{array}{l} \frac{dN}{dt}=r_{1}\left(1-{2\mu }_{1}\right)N-c\left(N^{2}+IN+EN\right),\\ \frac{dI}{dt}=\left(r_{2}-2r_{2}p_{e}-d\right)I+r_{1}\mu_{1}N+k\cdot BE-c\left(I^{2}+NI+EI\right),\\ \frac{dE}{dt}=r_{2}E+r_{2}p_{e}I-k\cdot BE-c\left(E^{2}+NE+IE\right).\; \end{array}\right.$$

The ODEs for the ICB therapy in the IHeM are provided below:

(6) $$\left\{\;\begin{array}{l} \frac{dN}{dt}=r_{1}\left(1-{2\mu }_{1}\right)N-c\left(N^{2}+AN+IN+EN\right),\\ \frac{dA}{dt}=\left(r_{1}-\mu_{2}f_{1}\right)A+r_{1}\mu_{1}N+\mu_{3}f_{2}I-c\left(A^{2}+NA+IA+EA\right),\\ \frac{dI}{dt}=\left(r_{2}-{2r}_{2}p_{e}-d\right)I-\mu_{3}f_{2}I+\mu_{2}f_{1}A+k\cdot BE-c\left(I^{2}+NI+AI+EI\right),\\ \frac{dE}{dt}=r_{2}E+r_{2}p_{e}I-k\cdot BE-c\left(E^{2}+NE+AE+IE\right).\; \end{array}\right.$$

We now incorporate chemotherapy into our model (Figure 2B). Acknowledging that faster proliferating cancer cells are generally more susceptible to chemotherapy [39, 40], we assume that NC (NCs) and ACs exhibit greater sensitivity to chemotherapy compared to ICs and escape cells (ECs). We introduce coefficients $K_{N}$, $K_{A}$, $K_{I}$, and $K_{E}$ to represent each cell type’s sensitivity to chemotherapy. Under chemotherapy, the death rate of neutral cancer cells is given by $r_{1}K_{N}\;\left(1-e^{-M}\right)$ [41]. The saturation term $1-e^{-M}$ characterizes the kill rate of chemotherapy: at relatively low concentrations of drug, the kill rate is nearly linear, whereas at higher drug concentrations, the kill rate plateaus. This form of kill rate is widely used to describe dose‐response curves and has been proven to yield good fits to in vitro data [33, 41, 42].

To align with findings suggesting that cancer cells overexpressing checkpoint ligands are less responsive to chemotherapy [29, 30], we set $K_{N}$ = $K_{A}$ = $K_{I}$ > $K_{E}$. Considering the known immunosuppressive effects of chemotherapies [33, 42, 43], we also incorporate immunotoxicity of chemotherapy into our models [44]. Let $K_{d}$ be the coefficient associated with chemotherapy‐induced damage to the immune system. We model this damage by adjusting the death rate as $d\;\to \;d\;\cdot \;\left(1\;–\;K_{d}\left(1\;–\;e^{-M}\;\right)\right)$.

Consequently, the ODEs describing chemotherapy for the IHoM are:

(7) $$\left\{\begin{array}{l} \frac{dN}{dt}=r_{1}\left(1-{2\mu }_{1}-K_{N}\;\left(1-e^{-M}\right)\right)N-c\left(N^{2}+IN+EN\right),\\ \frac{dI}{dt}=r_{2}\left(1-2p_{e}-K_{I}\;\left(1-e^{-M}\right)\right)I-d\cdot \left({1-K}_{d}\;\left(1-e^{-M}\right)\right)I+r_{1}\mu_{1}N\;\\ \;-c\left(I^{2}+NI+EI\right),\\ \frac{dE}{dt}=r_{2}{\left(1-K_{E}\;\left(1-e^{-M}\right)\right)E+r}_{2}p_{e}I-c\left(E^{2}+NE+IE\right), \end{array}\right.$$

For the IHeM, we have

(8) $$\left\{\begin{array}{l} \frac{dN}{dt}=r_{1}\left(1-{2\mu }_{1}-K_{N}\;\left(1-e^{-M}\right)\right)N-c\left(N^{2}+AN+IN+EN\right),\\ \frac{dA}{dt}=\left(r_{1}-\mu_{2}f_{1}-r_{1}K_{A}\;\left(1-e^{-M}\right)\right)A+r_{1}\mu_{1}N+\mu_{3}f_{2}I-c\left(A^{2}+NA+IA+EA\right),\\ \frac{dI}{dt}=r_{2}\left(1-2p_{e}-K_{I}\;\left(1-e^{-M}\right)\right)I-d\cdot \left({1-K}_{d}\;\left(1-e^{-M}\right)\right)I-\mu_{3}f_{2}I+\mu_{2}f_{1}A\;\\ \;-c\left(I^{2}+NI+AI+EI\right),\\ \frac{dE}{dt}=r_{2}{\left(1-K_{E}\;\left(1-e^{-M}\right)\right)E+r}_{2}p_{e}I-c\left(E^{2}+NE+AE+IE\right).\; \end{array}\right.$$

Combining the ICB and chemotherapy models mentioned above, we can establish a model for chemo‐ICB combination therapy as follows:

(9) $$\left\{\begin{array}{l} \frac{dN}{dt}=r_{1}\left(1-{2\mu }_{1}-K_{N}\;\left(1-e^{-M}\right)\right)N-c\left(N^{2}+AN+IN+EN\right),\\ \frac{dA}{dt}=\left(r_{1}-\mu_{2}f_{1}-r_{1}K_{A}\;\left(1-e^{-M}\right)\right)A+r_{1}\mu_{1}N+\mu_{3}f_{2}I-c\left(A^{2}+NA+IA+EA\right),\\ \frac{dI}{dt}=r_{2}\left(1-2p_{e}-K_{I}\;\left(1-e^{-M}\right)\right)I-d\cdot \left({1-K}_{d}\;\left(1-e^{-M}\right)\right)I-\mu_{3}f_{2}I+\mu_{2}f_{1}A\;\\ \;+k\cdot BE-c\left(I^{2}+NI+AI+EI\right),\\ \frac{dE}{dt}=r_{2}{\left(1-K_{E}\;\left(1-e^{-M}\right)\right)E+r}_{2}p_{e}I-k\cdot BE-c\left(E^{2}+NE+AE+IE\right).\; \end{array}\right.$$

### 2.3 Stochastic simulation of cancer evolution

To simulate the impact of intra‐tumoral heterogeneity on cancer treatment in greater detail and validate the ODEs model, we conduct stochastic simulations employing a stochastic branching process to depict antigen accumulation during cancer progression (Figure 1C). The general processes of the stochastic simulation are provided in Algorithm 1. All cellular processes align with the ODE models depicted in Figure 1A,B. Identical parameter sets are used in both simulations and the ODE models. Furthermore, we introduce cell competition in our simulations to regulate population sizes (see METHODS for details).

Algorithm 1. **Stochastic simulation algorithm for tumor growth and treatment**

1

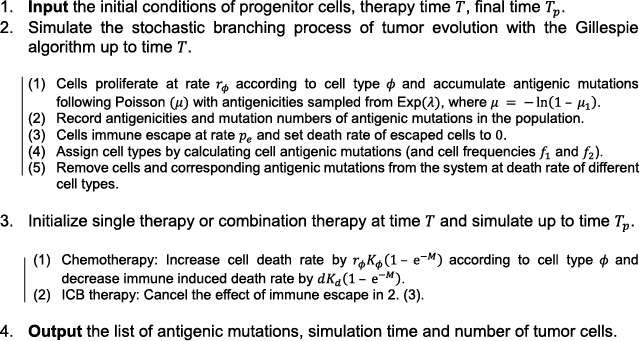

### 2.4 Evolutionary dynamics of IHoM and IHeM tumors

**FIGURE 3:**
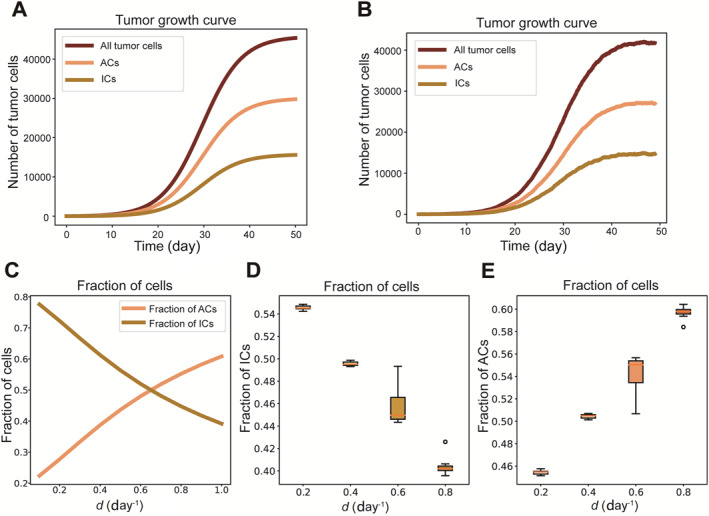
Growth dynamics of the IHeM. (A) Tumor growth curves generated with numerical solutions of ODEs of the IHeM. Growth curves of ACs, ICs and all tumor cells are shown. (B) Tumor growth curves generated with simulation data of ACs, ICs and all tumor cells in the IHeM. (C) Fractions of ACs and ICs generated with numerical solution of ODEs of the IHeM. (D) Box plots of fractions of ACs generated with 10 simulations of the stochastic IHeM. (E) Box plots of fractions of ICs generated with 10 simulations of the stochastic IHeM. Parameters: ($r_{1}$, $r_{2}$, $\mu_{1}$, $\mu_{2}$, $\mu_{3}$, d, c, $p_{e}$) = (1, 0.5, 0.5, 0.6, 0.05, 1, $1\times 10^{-5}$, 0). ACs, antigenic cells; ICs, immunogenic cells; IHeM, immunogenic heterogeneity model.

In this section, we uncover the differences in evolutionary dynamics between IHoM and IHeM by comparing the behavior of various cell components within the tumor under immune predation. We initially investigate the growth dynamics of IHeM by employing both ODEs and stochastic simulations. The corresponding parameter values are detailed in Table 1 (see METHODS). To study cancer growth in the absence of immune escape, we set $p_{e}=0$. Cancer progression attains equilibrium due to cell competition, as depicted in Figure 3A,B. To assess how the intrinsic strength of immune predation influences cancer, we subsequently examine cancer growth dynamics under the influence of the death rate d for ICs. Our findings indicate that an increase in d leads to a decrease in the proportion of ICs and an increase in the proportion of ACs, as illustrated in Figure 3C. Similar results are observed through stochastic simulations, as shown in Figure 3D and E. The intriguing dynamics observed at equilibrium, highlighting the proportions of antigenic and ICs, unveil how cancers adapt to auto‐immunity within the IHeM. This outcome also offers a plausible explanation for the immune system’s failure to eliminate heterogeneous cancers burdened with a significant antigen load [6, 7, 10]. This finding also indicates ICB therapy may fail in IHeM since it only reverses the effect of immune escape.

**TABLE 1:** Estimated parameter values.

Parameters	Description	Estimated value	References
$r_{1}$	Proliferation rate (NCs and ACs)	1	[34]
$r_{2}$	Proliferation rate (ICs and ECs)	0.5	
$\mu_{1}$	Antigenic mutation rate	0.5	[4, 6]
$\mu_{2}$	Immune recognition coefficient	0.6	
$\mu_{3}$	Effect of neoantigen heterogeneity	0.05	
d	Cell death rate	[0,1]	
c	Cell competition death rate	$10^{-8}$	
$p_{e}$	Immune escape rate	$10^{-5}$	
f	Treatment frequency coefficient	2π/21	[4, 34]
γ	Rate of drug decay	0.9	[16]
$K_{N}\;{,K}_{A}\;{,K}_{I}$	Fractional cell kill by chemotherapy	0.9	[33, 45]
$K_{E},K_{d}$	Fractional immune cell kill by chemotherapy	0.6	[33, 46]
$v_{m}$	Dose strength of chemotherapy drug	[1,5]	[33, 46]
$v_{b}$	Dose strength of ICB therapy drug	[0.1,0.9]	[33]
k	Efficacy of ICB therapy drug	1	

**FIGURE 4:**
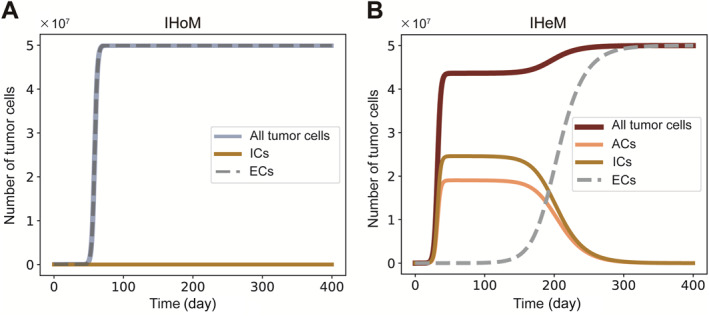
Comparison of the IHoM and IHeM. (A) Growth curves generated from the numerical solutions of ODEs of the IHoM. (B) Growth curves generated from the numerical solutions of ODEs of the IHeM. Growth curves of all types of cells are shown. Parameters: ($r_{1}$, $r_{2}$, $\mu_{1}$, $\mu_{2}$, $\mu_{3}$, d, c, $p_{e}$) = (1, 0.5, 0.5, 0.6, 0.05, 1, $1\times 10^{-8}$, $1\times 10^{-5}$). IHeM, immunogenic heterogeneity model; IHoM, immunogenic homogeneity model.

To account for cancer treatment, we introduce immune escape into our models ($p_{e}\;>\;0$). Our findings reveal that tumors undergo population shifts toward immune‐escaped cells, ultimately reaching equilibrium (Figure 4). It is worth noting that immune escape occurs early in tumors under the IHoM and later in those under the IHeM (Figure 4). The tumors in these two models follow distinct evolutionary paths before achieving complete immune escape at the whole tumor level. In the IHoM, tumors are swiftly dominated by immune‐escaped cells derived from ICs, establishing immune escape as the primary mechanism promoting tumor survival. Conversely, in the IHeM, tumors exhibit a capacity for immune selection evasion through high heterogeneity. The significant immune escape observed in hypermutable tumors is considered vital for achieving tumor homogeneity and eliciting an effective immunotherapy response [15, 36]. Nevertheless, our results do not indicate a clear distinction between the IHoM and the IHeM based on this criterion.

### 2.5 Chemotherapy creates tumor immunological conditions favorable for ICB

**FIGURE 5:**
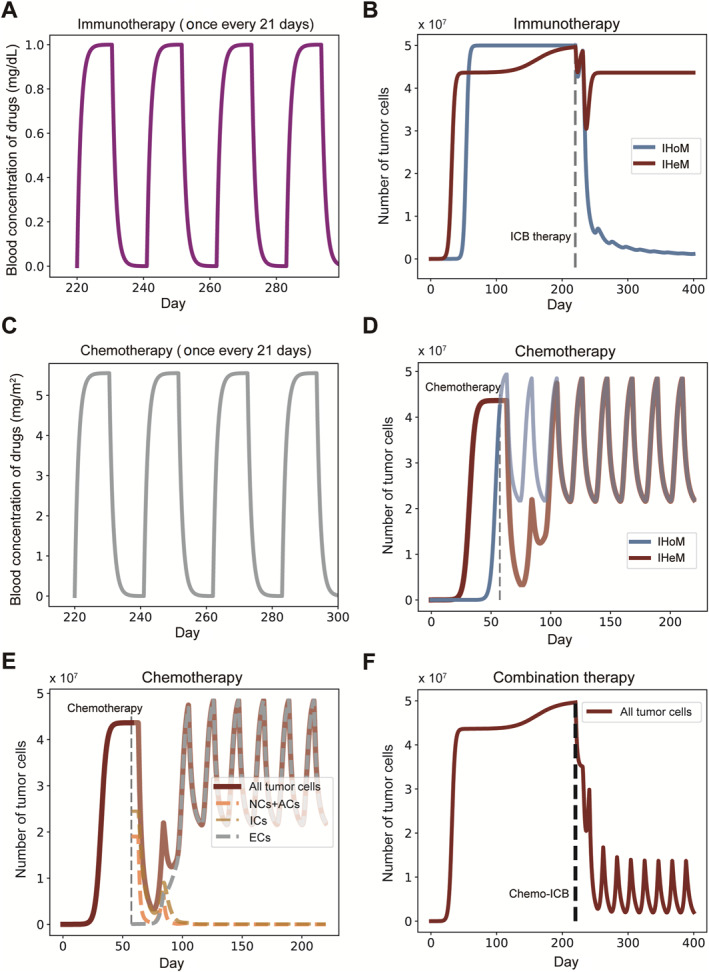
Responses to different therapies. (A) Tumor growth curves under ICB therapy. (B) Blood concentration change of ICB drug corresponding to treatment cycle. (C) Tumor growth curves under chemotherapy. (D) Blood concentration change of chemotherapy drug corresponding to treatment cycle. (E) Growth curves of (NCs + ACs), ICs and ECs under chemotherapy generated with numerical solutions. (F) Tumor growth curves under combination therapy. Parameters: ($r_{1}$, $r_{2}$, $\mu_{1}$, $\mu_{2}$, $\mu_{3}$, d, c, $p_{e}$, f, γ, $K_{N}$, $K_{A}$, $K_{I}$, $K_{E}$, $K_{d}$, $v_{m}$, $v_{b}$) = (1, 0.5, 0.5, 0.6, 0.05, 0.5, $1\times 10^{-8}$, $1\times 10^{-5}$, $\frac{2\pi }{21}$, 0.9, 0.9, 0.9, 0.9, 0.6, 0.6, 5, 0.9). ACs, antigenic cells; ICB, immune checkpoint blockade; ICs, immunogenic cells; NCs, Neutral Cells.

To reveal the governing mechanisms of chemo‐ICB therapy response, we study the dynamics of cancers under different therapies with ODE models and stochastic simulations. We investigate the impact of ICB and chemotherapy, utilizing standard chemotherapy protocols with a 21‐day treatment cycle. Following the conventional practice where drugs are administered during the first half of the cycle, allowing the latter half for patient recovery [16], both chemotherapy and ICB are administered on the same day [47]. Each treatment cycle consists of 21 days with one pulse of drugs administered (Figure 5A,C).

Initially, we examine the response of tumors under each model to single therapy. The results indicate that the tumor under the IHoM is responsive to ICB, leading to its extinction, whereas the tumor under the IHeM remains unresponsive, maintaining a high equilibrium population size (Figure 5B). This outcome is consistent with previous findings [6, 7, 8]. In contrast to the IHoM, the tumor under the IHeM exhibits an early response followed by rapid relapse when treated with chemotherapy under the same strategy (Figure 5D). To elucidate this phenomenon in the IHeM, we examine the dynamics of various cell components within the tumor. We observe a rapid increase in immune‐escaped cells (ECs) after the initial response (Figure 5E). The heightened sensitivity of non‐cancerous cells (NCs) and ACs to chemotherapy leads to their rapid decrease and extinction. Simultaneously, chemotherapy‐induced damage to the immune system facilitates immune escape, providing ECs with a distinct survival advantage and resulting in rapid relapse.

Following the relapse, the tumor, initially evolving under the dynamics of the IHeM, now transitions to being governed by the IHoM. This transition results in a curative effect when combined with ICB therapy (Figure 5F). This outcome aligns with previous findings suggesting that chemotherapy increases PD‐L1 expression in cancers [48]. We have elucidated how chemotherapy can induce favorable immunological conditions in chemo‐ICB combination therapy, emphasizing the significant responsiveness of the IHoM to ICB.

**FIGURE 6:**
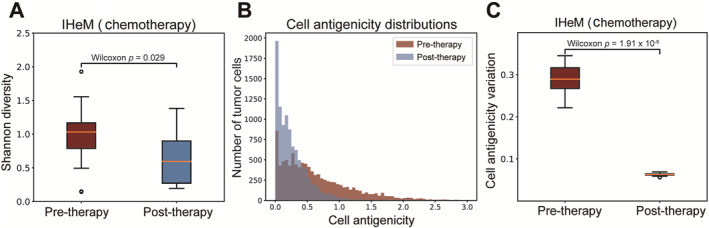
Effect of chemotherapy on antigens diversity. (A) Box plots of Shannon diversities of neoantigens before and after 10 simulated chemotherapies. (B) Distribution of cell antigenicity before and after one simulated chemotherapy. (C) Box plots of cell antigenicity variation before and after 10 simulated chemotherapies.

To validate the transformation of the IHeM to the IHoM during early remission under chemotherapy, we examine tumor antigenic heterogeneity before and after chemotherapy. Referring to Figure 4B, we observe that remission under chemotherapy occurs before tumor immune escape. We simulate chemotherapy and record the neoantigen landscapes of the tumors. Cell antigenicity, defined as the sum of all neoantigens’ antigenicity for each cell, serves as our metric. The simulation results demonstrate a decline in tumor antigenic Shannon diversity after chemotherapy (Figure 6A). Additionally, the variations in tumor cell antigenicity distributions are significantly reduced post‐chemotherapy (Figure 6B,C). These findings suggest that tumors become more homogeneous after chemotherapy. Given that studies indicate antigenically homogeneous tumors are responsive to ICB therapy [6, 7, 49], our results unveil the underlying mechanism governing the response to ICB in combination with chemotherapy.

### 2.6 Immune recovery is crucial for enhancing combination therapy response

**FIGURE 7:**
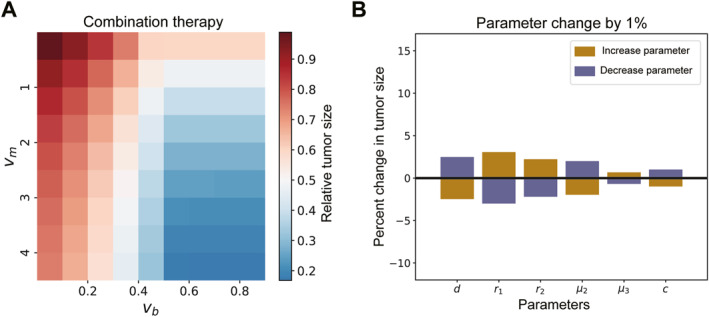
Effect of drug dose pair and sensitivity analysis. (A) Therapeutic effect of different dose pairs of ICB and chemotherapy drugs. Therapeutic effect measured by relative tumor size (Post therapy population size/Pre‐therapy population size). (B) Sensitivity analysis on parameters. ICB, immune checkpoint blockade.

In this section, we aim to study the role of immune recovery by evaluating the therapeutic effects under different drug dose pairs and presenting a treatment protocol considering alternate medication. The therapeutic response to drug combinations often improves with higher doses, but the consideration of patient tolerance during treatment is crucial. To explore the therapeutic effect of different dose pairs of drugs, we calculate the relative tumor size under each dose pair. It is worth noting that a small dose of chemotherapy drug ($v_{m}\leq 1$) combined with a certain ICB dose exceeding 0.5 demonstrate a significant therapeutic response (Figure 7A). The use of a smaller chemotherapy dose causes less damage to the immune system while still modifying the tumor’s immunological conditions, thus showing promising effects in combination with ICB. This result underscores the pivotal role that the immune system plays in combination therapy. Utilizing parameter values from Table 1, we conduct sensitivity analysis, revealing that our model results are not highly sensitive to changes in parameter values (Figure 7B). This outcome validates the robustness of our conclusions and reinforces the reliability of our model’s predictions.

**FIGURE 8:**
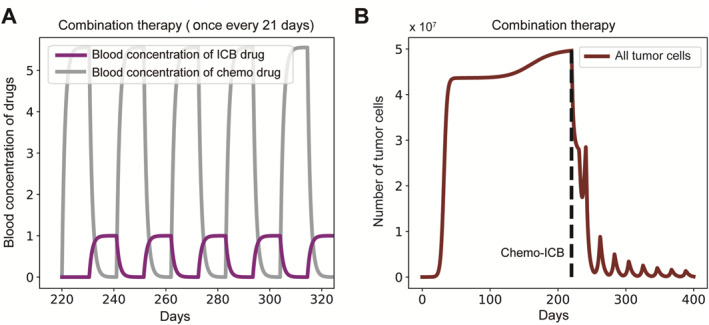
Revised combination therapy. (A) Blood concentration curve of revised therapy protocol. (B) Tumor growth curves under combination therapy under revised therapy protocol. Parameters: ($r_{1}$, $r_{2}$, $\mu_{1}$, $\mu_{2}$, $\mu_{3}$, d, c, $p_{e}$, f, γ, $K_{N}$, $K_{A}$, $K_{I}$, $K_{E}$, $K_{d}$, $v_{m}$, $v_{b}$, h) = (1, 0.5, 0.5, 0.6, 0.05, 0.5, $1\times 10^{-8}$, $1\times 10^{-5}$, $\frac{2\pi }{21}$, 0.9, 0.9, 0.9, 0.9, 0.6, 0.6, 5, 0.9, 0.5).

Based on the aforementioned results, the detrimental impact of chemotherapy on the immune system significantly influences the effectiveness of combination therapy. To mitigate the damage caused by chemotherapy to the immune system, we explore an alternative treatment protocol. In this revised approach, tumors are treated with chemotherapy during the first half of each treatment cycle, followed by ICB therapy in the second half (Figure 8A). We assume that this modified therapy protocol results in less immune system damage, introducing a healing factor h. Consequently, we adjust the immune cell death rate from $d\;\cdot \;\left(1\;–\;K_{d}\;\left(1\;–\;e^{-M}\;\right)\right)$ to $d\;\cdot \;\left(1-h\;\cdot \;K_{d}\;\left(1\;–\;e^{-M}\right)\right)$, resulting in an improved combination therapy outcome (Figure 8B). This restructured treatment approach leads to a mitigated relapse in each treatment cycle, ultimately culminating in the successful cure of cancer. Our findings suggest that even when the immune‐damaging effects of chemotherapy may diminish the effectiveness of ICB treatment, maintaining the order of medication—chemotherapy preceding ICB treatment—remains a favorable strategy.

## 3 DISCUSSIONS

In this study, we investigate the dynamics of cancer evolution during chemo‐ICB therapy through the application of mathematical models. The adaptability of cancers to the immune system has been explored through various mechanisms [50, 51, 52]. By using our models, we illustrate an immune escape mechanism observed in antigenically heterogeneous tumors. This escape mechanism operates by regulating the proportions of ACs and ICs in a frequency‐dependent manner. The presence of such a mechanism aligns with reported instances of treatment failure in ICB, particularly in tumors characterized by a high burden of antigenic mutations that remain unresponsive to ICB [53]. Our assumptions, positing the origin of cancers from NC with subsequent differentiation into ICs [35, 54, 55, 56], allow us to explain how chemotherapy creates a conducive environment for tumor growth under immunogenic conditions. Despite the potential immune system damage caused by chemotherapy [33, 42, 43], an intriguing trade‐off arises. This trade‐off involves the transformation of the IHeM to the IHoM, ultimately rendering the tumor more amenable to ICB therapy. Our findings also highlight the enhanced therapy response observed in combination therapy scenarios. Furthermore, we conduct an analysis of prognostic factors such as cell proliferation rate and drug doses, shedding light on their influences within the context of the modeled therapeutic approaches.

The classical predator–prey model framework applied to cancer not only considers the growth dynamics of tumor cells but also account for the dynamics of effector cells in the tumor microenvironment, including T cells, NK cells, and Tregs cells, among others [33]. In our mathematical models, we quantify the impact of the tumor microenvironment using parameters, focusing on the intra‐tumoral evolutionary mechanisms and characterizing the growth dynamics within the tumor‐immune microenvironment. We present mutation events such as antigenic mutation with the form of NC → IC in our models. Antigenic mutation can be presented as both NC → NC + IC and NC → IC + IC. Since antigenic mutation accumulation is a stochastic event, it is comprehensive to include both symmetric cell division (NC → IC + IC) and asymmetric cell division (NC → NC + IC). However, these processes result in additional parameters, which may cause some identifiability issues during parameter estimation, and we have not found reasonable ranges for the relevant parameters in the previous literature. We think that NC → IC is a reasonable simplification of those processes as the core cellular changes in the IHoM model are cell proliferation and mutation. Moreover, our results reveal the significance of the tumor‐immune microenvironment, particularly under the influence of chemotherapy. In terms of pharmacological toxicity, recent research has demonstrated that maintaining low concentrations of chemotherapeutic drugs and relatively high concentrations of immunotherapy drugs can effectively mitigate toxic effects [57], aligning with our own findings.

Our models consider the combination of chemotherapy and ICB therapy; however, the design of combination therapy typically involves selecting the optimal drug combination, determining the best dose pair, and establishing the order of drug delivery [24, 25, 58, 59]. Although our models clarify general mechanisms underpinning therapies, we acknowledge that the intricacies of combination therapy design are multifaceted. We use parameters sourced from existing literature [4, 6, 16, 33, 34, 45, 46]. However, we encounter challenges in fitting our modeling results to actual patient data. Our models are constructed based on the assumption of cellular hierarchy and its applications to tumor immunology. Initially, our model assume that ACs and ICs follow distinct hierarchies of cell differentiation. However, real‐world tumor scenarios may be more complex. We discuss the implications if the actual situation diverges from our hypothesis (Figure S1). It is worth noting that as the proliferation rate of ACs diminishes relative to that of ICs, the differences in responses between ICB and chemotherapy decrease (Figure S1A,B). Furthermore, responses to combination therapy decline as ACs become more differentiated than ICs (Figure S1C). Despite these considerations, combination therapy still demonstrates an improved response compared to single treatments, underscoring its potential efficacy across various proliferation rate scenarios.

By uncovering general mechanisms of cancer evolution under therapeutic interventions, our models yield crucial insights for optimizing combination therapy strategies. The integration of chemotherapy and immunotherapy represents a promising avenue in cancer treatment, demonstrating positive outcomes in clinical trials. In practical application of our framework, one can use a predictive biomarker to assess tumor antigenic heterogeneity [35]. The next frontier involves refining chemo‐ICB regimens based on synergistic mechanisms to enhance clinical efficacy and developing better biomarkers that help in evaluating tumor heterogeneity. Our models provide a framework for integrating drug combinations with patient data in future applications. Additionally, the optimal drug delivery order in combination alternate therapy is a pivotal consideration in treatment strategy optimization. Our work addresses this question, asserting that the order of medication administration should prioritize chemotherapy before ICB treatment. This finding contributes valuable guidance to the ongoing efforts in tailoring effective combination therapies for cancer patients.

## 4 METHODS

### 4.1 The derivation of model equations

In the IHoM, we generate stochastic master equation for the dynamics illustrated in Figure 1A. Let $N_{t}$, $A_{t}$, $I_{t}$, and $E_{t}$ be the cell numbers of NCs, ACs, ICs, and ECs at time t, respectively. The stochastic dynamics of IHoM are captured by the following master equation:

(10) $$\frac{\partial \phi \left(N_{t},I_{t},E_{t}\right)}{\partial t}=r_{1}\left(1-\mu_{1}\right)\left(N_{t}-1\right)\phi \left(N_{t}-1,I_{t},E_{t}\right)+r_{1}\mu_{1}\left(N_{t}+1\right)\phi \left(N_{t}+1,I_{t}-1,E_{t}\right)+r_{2}\left(1-p_{e}\right)\left(I_{t}-1\right)\phi \left(N_{t},I_{t}-1,E_{t}\right)+r_{2}p_{e}\left(I_{t}+1\right)\phi \left(N_{t},I_{t}+1,E_{t}-1\right)A+r_{2}\left(E-1\right)\phi \left(N_{t},I_{t},E_{t}-1\right)+d\left(I_{t}+1\right)\phi \left(N_{t},I_{t}+1,E_{t}\right)+c\left(N_{t}+1\right)\left(N_{t}+I_{t}+E_{t}\right)\phi \left(N_{t}+1,I_{t},E_{t}\right)+c\left(I_{t}+1\right)\left(N_{t}+I_{t}+E_{t}\right)\phi \left(N_{t},I_{t}+1,E_{t}\right)+c\left(E_{t}+1\right)\left(N_{t}+I_{t}+E_{t}\right)\phi \left(N_{t},I_{t},E_{t}+1\right)-\phi \left(N_{t},I_{t},E_{t}\right)(r_{1}N_{t}+r_{2}I_{t}+r_{2}E_{t}+dI_{t\;}+c\left(N_{t}-1\right)\left(N_{t}+I_{t}+E_{t}\right)+c\left(I_{t}-1\right)\left(N_{t}+I_{t}+E_{t}\right)+c\left(E_{t}-1\right)\left(N_{t}+I_{t}+E_{t}\right))$$

Let $\phi \left(N,I,E\right):=P\;\left(N_{t}=N,I_{t}=I,E_{t}=E\right)$, representing the joint probability distribution of $\left(N_{t},I_{t},E_{t}\right)$. For simplicity, we use (N,I,E) to denote $\left(N_{t},I_{t},E_{t}\right)$. Let 〈·〉 denote the expectation of random variables N, I, and E. We have 〈N〉, 〈I〉, and 〈E〉 representing the expectations of NCs, ICs, and ECs. For 〈N〉, observing that $\langle N\rangle =\sum_{N,I,E}N\;\phi \left(N,I,E\right)$, we multiply the master Equation (10) by N and then sum over N, I, and E. We obtain the following:

$$\frac{d\langle N\rangle }{dt}=r_{1}\left(1-\mu_{1}\right)\langle N^{2}\rangle +r_{1}\left(1-\mu_{1}\right)\langle N\rangle +r_{1}\mu_{1}\langle N^{2}\rangle -r_{1}\mu_{1}\langle N\rangle +r_{2}\left(1-p_{e}\right)\langle NI\rangle +{r_{2}p_{e}\langle NI\rangle +r}_{2}\langle NE\rangle +d\langle NI\rangle -\left(r_{1}\langle N^{2}\rangle +r_{2}\langle NI\rangle +r_{2}\langle NE\rangle +d\langle NI\rangle \right)-c\left(\langle N^{2}\rangle +\langle NI\rangle +\langle NE\rangle \right)=r_{1}\left(1-2\mu_{1}\right)\langle N\rangle -c\left(\langle N^{2}\rangle +\langle NI\rangle +\langle NE\rangle \right)$$

Similarly, for 〈I〉 and 〈E〉 we have

$$\frac{d\langle I\rangle }{dt}=\left(r_{2}-r_{2}p_{e}\right)\langle I\rangle +r_{1}\mu_{1}\langle N\rangle -r_{2}p_{e}\langle I\rangle -d\langle I\rangle -c(\langle I^{2}\rangle +\langle NI\rangle +\langle EI\rangle )$$

and

$$\frac{d\;\langle E\rangle }{dt}=r_{2}\;\langle E\rangle +r_{2}p_{e}\;\langle I\rangle -c(\langle E^{2}\rangle +\langle NE\rangle +\langle IE\rangle ).$$

With ODEs of 〈N〉, 〈I〉, and 〈E〉, we remove the sign 〈·〉 and derive model equations (1) for IHoM in the main text.

For the IHeM, the stochastic dynamics of IHeM are captured by the following master equation:

(11) $$\frac{\partial \phi \left(N_{t},A_{t},I_{t},E_{t}\right)}{\partial t}=r_{1}\left(1-\mu_{1}\right)\left(N_{t}-1\right)\phi \left(N_{t}-1,A_{t},I_{t},E_{t}\right)+r_{1}\left(A_{t}-1\right)\phi \left(N_{t},A_{t}-1,I_{t},E_{t}\right)+r_{1}\mu_{1}\left(N_{t}+1\right)\phi \left(N_{t}+1,{A_{t}-1,I}_{t},E_{t}\right)+\mu_{2}f_{1}\left(A_{t}+1\right)\phi \left(N_{t},A_{t}+1,I_{t}-1,E_{t}\right){+\mu }_{3}f_{2}\left(I_{t}+1\right)\phi \left(N_{t},A_{t}-1,I_{t}+1,E_{t}\right)+r_{2}\left(1-p_{e}\right)\left(I_{t}-1\right)\phi \left(N_{t},A_{t},I_{t}-1,E_{t}\right){+r}_{2}p_{e}\left(I_{t}+1\right)\phi \left(N_{t},{A_{t},I}_{t}+1,E_{t}-1\right)+r_{2}\left(E-1\right)\phi \left(N_{t},A_{t},I_{t},E_{t}-1\right)+d\left(I_{t}+1\right)\phi \left(N_{t},A_{t},I_{t}+1,E_{t}\right)+\;c\left(N_{t}+1\right)\left(N_{t}+A_{t}+I_{t}+E_{t}\right)\phi \left(N_{t}+1,A_{t},I_{t},E_{t}\right)+c\left(A_{t}+1\right)\left(N_{t}+A_{t}+I_{t}+E_{t}\right)\phi \left(N_{t},A_{t}+1,I_{t},E_{t}\right)+c\left(I_{t}+1\right)\left(N_{t}+A_{t}+I_{t}+E_{t}\right)\phi \left(N_{t},A_{t},I_{t}+1,E_{t}\right)+c\left(E_{t}+1\right)\left(N_{t}+A_{t}+I_{t}+E_{t}\right)\phi \left(N_{t},A_{t},I_{t},E_{t}+1\right)-\phi \left(N_{t},A_{t},I_{t},E_{t}\right)(\;r_{1}N_{t}+{r_{1}A_{t}+\mu_{2}f_{1}A_{t}+r}_{2}I_{t}+\mu_{3}f_{2}I_{t}+r_{2}E_{t}+dI_{t}+c\left(N_{t}-1\right)\left(N_{t}+A_{t}+I_{t}+E_{t}\right)+c\left(A_{t}-1\right)\left(N_{t}+A_{t}+I_{t}+E_{t}\right)+c\left(I_{t}-1\right)\left(N_{t}+A_{t}+I_{t}+E_{t}\right)+c\left(E_{t}-1\right)\left(N_{t}+A_{t}+I_{t}+E_{t}\right))$$

Similarly, we derive model Equation (2) for IHeM according to Equation (11) in the main text.

To our knowledge, antigenic mutation can be presented as both NC → NC + IC and NC → IC + IC. Since antigenic mutation accumulation is a stochastic event, it is comprehensive to include both symmetric cell division (NC → IC + IC) and asymmetric cell division (NC → NC + IC). Noting that immune escape also comes along with cell division, it can be presented as IC → IC + EC and IC → EC + EC. We can characterize the schematic representation of a full model (IHoM) as follows:

(a) NC $\overset{r_{1}\left(1-\mu_{1}\right)}{\to }$ NC + NC,
(b) NC $\overset{r_{1}\mu_{11}}{\to }$ NC + IC,
(c) NC $\overset{r_{1}\mu_{12}}{\to }$ IC + IC,
(d) IC $\overset{r_{2}\left(1-p_{e}\right)}{\to }$ IC + IC,
(e) IC $\overset{r_{2}p_{e_{1}}}{\to }$ IC + EC,
(f) IC $\overset{r_{2}p_{e_{2}}}{\to }$ EC + EC,
(g) IC $\overset{d}{\to }\emptyset$,
(h) EC $\overset{r_{2}}{\to }$ EC + EC.

where $\mu_{1}=\mu_{11}+\mu_{12}$ and $p_{e}=p_{e_{1}}+p_{e_{2}}$. We derive model equations as follows:

(12) $$\left\{\begin{array}{l} \frac{dN}{dt}=r_{1}\left(1-\mu_{1}\right)N-r_{1}\mu_{12}N-c\left(N^{2}+IN+EN\right),\\ \frac{dI}{dt}=\left(r_{2}-r_{2}p_{e}\right)I+\left(r_{1}\mu_{11}+{2r}_{1}\mu_{12}\right)N-r_{2}p_{e_{2}}I-dI-c\left(I^{2}+NI+EI\right),\\ \frac{dE}{dt}=r_{2}E+\left(r_{2}p_{e_{1}}+2r_{2}p_{e_{2}}\right)I-c\left(E^{2}+NE+IE\right).\; \end{array}\right.$$

We find that, in terms of form, the terms in the two sets of equations are consistent, with the difference being that Equation (12) has more parameters. These additional parameters may cause some identifiability issues during parameter estimation, and we have not found reasonable ranges for the relevant parameters in the previous literature. We think that Equation (1) is a reasonable simplification of Equation (12), as the core cellular changes in the IHoM model are cell proliferation and mutation. Although Equation (1) is not as comprehensive as the Equation (12) model, it provides a good representation of both cell proliferation and mutation processes through symmetric division and cell state transitions.

### 4.2 Parameter estimation

The parameter values used in the analysis are identical for tumor progression and tumors under therapies. We have estimated suitable ranges for parameter values from the published literature. For those that could not be found in the literature, we try to delineate a reasonable range for parameter values by accessing their biological meanings and associations with other parameters. Specifically, parameter $r_{2}$ represents tumor cell proliferation rate of ICs and ECs, therefore, we have $0\leq r_{2}\leq 1$. Similarly, it is reasonable to have: 0≤d≤1. Since ICs and ECs are originated from NCs and ACs, $r_{2}$ is generally set to be no greater than $r_{1}$. To delineate the dynamics in highly immunogenic tumors, we assume that over a half of ACs can transform into ICs, thus we set $\mu_{2}=0.6$. Due to tumor antigenic heterogeneity, $\mu_{3}$ is used to characterize immune recognition capacity causing a small portion of ICs to be prevented from immune predation. Therefore, we set $\mu_{3}$ to be much smaller than $\mu_{1}$ and $\mu_{2}$. According to the suggested serum concentration in Ref. [60] and the biological fact that $v_{b}$ influences the rate of ECs converting to ICs during ICB therapy, it is also set to be within the range of [0,1]. We fix $K_{E}$ value to equal to $K_{d}$ to align with EC’s insensitivity to chemotherapy. Since our aim is to study the underlying mechanism of combination therapy, we set the efficacy of ICB drug k to be 1 to represent enough effect of ICB therapy. All parameters are summarized in Table 1.

### 4.3 Stochastic simulation of cancer evolution

We conduct stochastic simulations employing a stochastic branching process to depict antigen accumulation during cancer progression (Figure 1C). The general processes of the stochastic simulation are provided in Algorithm 1. In each simulation step, cells that are antigenically neutral and belong to a lineage can proliferate at a rate of $r_{1}$. The two daughter cells resulting from each division accumulate antigenic mutations at an overall rate of μ. The number of generated mutations, denoted as m, is sampled from a Poisson distribution (Poisson (μ)), and each antigen’s antigenicity is drawn from an exponential distribution (Exp (λ) where λ=0.2) [34]. In the IHoM, when a cell acquires an antigenic mutation, it transforms into an IC, whereas in the IHeM, it becomes an AC. ACs can proliferate at a rate of $r_{1}$, and ICs can proliferate at a rate of $r_{2}$. To ensure parameter consistency between the ODEs and stochastic simulations, we select parameters such that $P\;\left(m\geq 1\right)=\mu_{1}$, where P(m≥1) represents the probability of cells acquiring no less than one neoantigen at cell division (m∼ Poisson (μ)), thus we have $1\;–\;e^{-\mu }=\mu_{1}$ and then $\mu =-\ln\left(1\;–\;\mu_{1}\right)$. ICs have a death rate d and an immune escape probability $p_{e}$. All cellular processes align with the ODE models depicted in Figure 1A and B. Identical parameter sets are used in both simulations and the ODE models. Furthermore, we introduce cell competition in our simulations to regulate population sizes.

## AUTHOR CONTRIBUTIONS

**Shaoqing Chen:** Conceptualization; formal analysis; funding acquisition; methodology; software; visualization; writing—original draft; writing—review and editing. **Zheng Hu:** Conceptualization; funding acquisition; methodology; supervision; writing—review and editing. **Da Zhou:** Conceptualization; funding acquisition; methodology; supervision; writing—original draft; writing—review and editing.

## CONFLICT OF INTEREST STATEMENT

The authors declare no conflicts of interest.

## ETHICS STATEMENT

This article does not involve any studies with human or animal subjects conducted by any of the authors.

## Supporting information

Figure S1

## Data Availability

The codes of model simulation are available at GitHub (github.com/Shaoqing1117/CheoICBSimulation).
