# Supplementary material for: Modeling combination chemo‐immunotherapy for heterogeneous tumors
Source: Quant Biol. 2025 Mar 14;13(3):e98. doi: 10.1002/qub2.98 (PMC12806026; doi:10.1002/qub2.98)
Supplement: Supplementary file 1 — Figure S1 [file QUB2-13-e98-s001.docx]

**SUPPLEMENTATY MATERIALS**


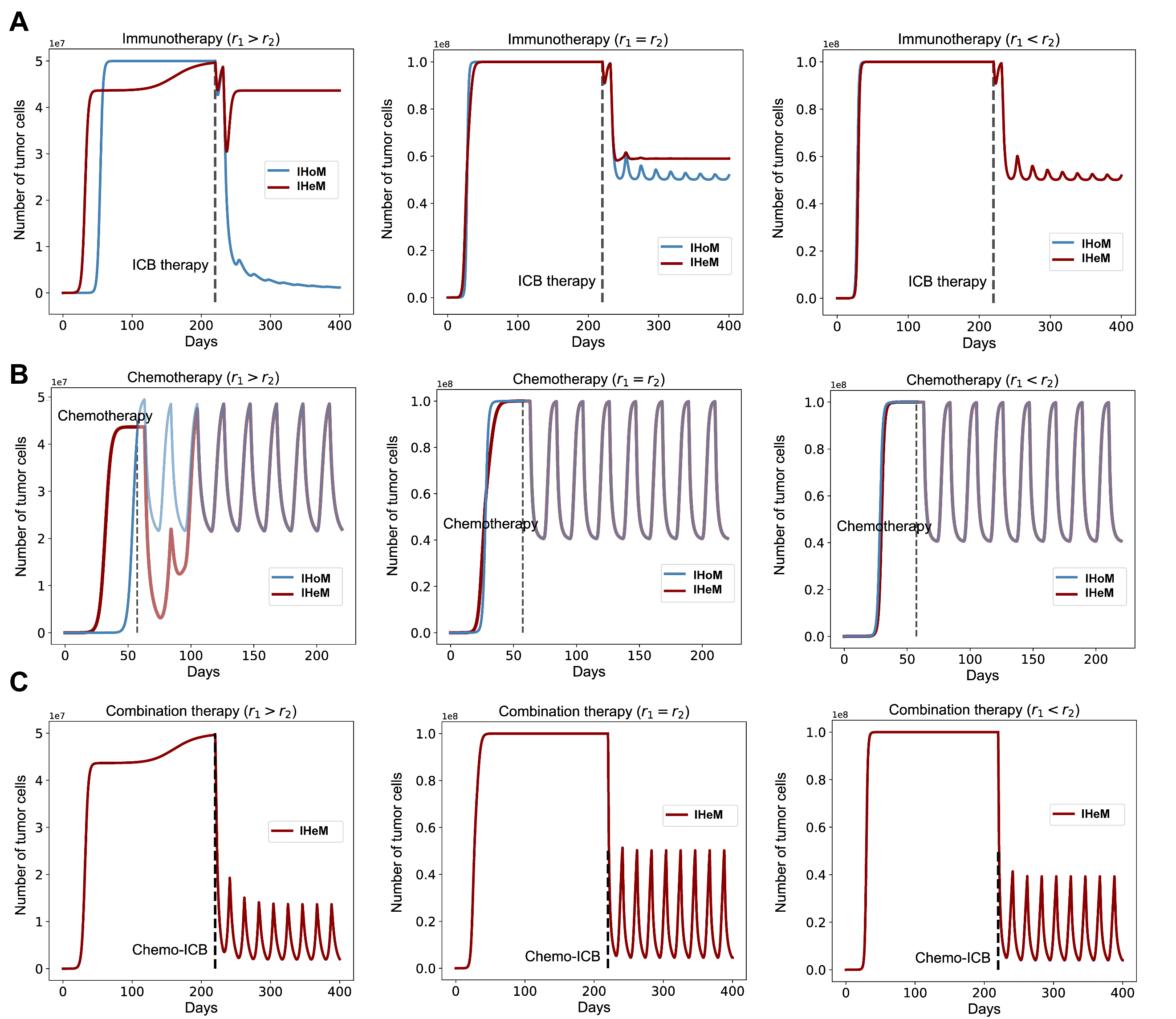


**Figure S1. Responses to therapies in different scenarios. A,** Tumor growth curves under ICB therapy. **B,** Tumor growth curves under chemotherapy. **C,** Tumor growth curves under combination therapy. Parameters: ($r_{1}$, $r_{2}$, $\mu_{1}$, $\mu_{2}$, $\mu_{3}$, $d$, $c$, $p_{e}$, $f$, $\gamma$, $K_{N}$ , $K_{A}$, $K_{I}$ , $K_{E}$,$K_{d}$, $v_{m}$, $v_{b}$) = ($1$, $0.5$, $0.5$, $0.6$, $0.05$, $0.5$, $1 \times{10}^{-8}$ , $1 \times{10}^{-5}$ , $\frac{2\pi}{21}$, $0.9$, $0.9$, $0.9$, $0.9$, $0.6$, $0.6$, $5$, $0.9$).$r_{1}> r_{2}$ : $\left( r_{1}, r_{2} \right)= \left( 1, 0.5 \right)$, $r_{1}= r_{2} : \left( r_{1}, r_{2} \right)= \left( 1, 1 \right)$, $r_{1}< r_{2} : \left( r_{1}, r_{2} \right)= \left( 0.5, 1 \right)$.
